# Generation of new hair cells by DNA methyltransferase (Dnmt) inhibitor 5-azacytidine in a chemically-deafened mouse model

**DOI:** 10.1038/s41598-019-44313-0

**Published:** 2019-05-29

**Authors:** Xin Deng, Zhenjie Liu, Xiaoyang Li, Yang Zhou, Zhengqing Hu

**Affiliations:** 1 Department of Otolaryngology-Head and Neck Surgery, Wayne State University School of Medicine, Detroit, USA; 2 John D. Dingell VA Medical Center, Detroit, Michigan USA

**Keywords:** Hair cell, Regeneration

## Abstract

Regeneration of mature mammalian inner ear hair cells remains to be a challenge. This study aims to evaluate the ability of DNA methyltransferase (Dnmt) inhibitor 5-azacytidine (5-aza) to generate outer hair cells (OHCs) in a chemically-deafened adult mouse model. 5-aza was administrated into the mouse inner ear via the round window. Immunofluorescence was used to examine the expression of hair cell specific proteins following 5-aza treatment. The results showed that in the chemically-deafened mouse cochlea, new OHCs were found post 5-aza treatment, whereas OHCs were completely lost in saline-treated mice. New hair cells expressed multiple hair cell markers included Myosin VIIa, Pou4f3 and Myosin VI. Newly-generated hair cells presented in three cochlear turns and were able to survive for at least six weeks. The effects of new hair cells generation by 5-aza were concentration dependent. Quantitative PCR study indicates that 5-aza may function through Dnmt1 inhibition. The results of this report suggest that the Dnmt inhibitor 5-aza may promote hair cell regeneration in a chemically-deafened mouse model.

## Introduction

Mammalian sensory hair cells consist of one row of inner hair cells (IHCs) and three rows of outer hair cells (OHCs) localizing in the organ of Corti above the basilar membrane in the inner ear. IHCs detect the sound signals from the outer ear and relay them to the brainstem through the auditory nerve, while OHCs amplify sound information^1^. Human auditory hair cell loss causes hearing loss that affects about 10% of the population. Hair cell degeneration can be caused by acoustic trauma, ototoxic drugs, aging, environmental or genetic influences^2^. In the mammalian auditory system, the regenerative ability of hair cells is limited within a couple of weeks after birth, and the regenerative capability of hair cells diminishes rapidly during maturation^3^.

Several approaches including gene therapy, cell transplantation and pharmacotherapy have been explored for hair cell regeneration^4^. The gene therapy allows for the introduction of transgenes to cochlear cells. For instance, overexpressing of *Atoh1* in mammalian inner ear results in new hair cells in neonatal and mature cochlear explants^5,6^. The stem cell-based transplantation takes advantage of the self-renewal and differentiation abilities of stem cells^4,7^. In terms of pharmacotherapy, it is found that antioxidants (e.g., N-acetyl-L-cysteine; NAC) and growth factors (e.g., neurotrophins) exhibit protection roles in preventing hearing loss^4,8^. However, most of these studies are performed using *in vitro* cochlear explants. In the *in vivo* studies, it is observed that *Lgr5*-positive supporting cells are able to become new hair cells in damaged neonatal mouse cochleae^9^. In another study where the hair cells of adult mouse cochlea are degenerated by acoustic trauma, new hair cells resulted from supporting cell transdifferentiation are generated by inhibition of the Notch signaling^10^. Wnt signaling pathway regulates the development and maturation of the mouse cochlea^11,12^. It is shown that inhibition of the Wnt signaling diminishes hair cell regeneration in neonatal mice^13^. These genetic approaches focus on specific genes and signaling pathways in hair cell regeneration, while the epigenetic approach is a largely understudied area.

Epigenetic regulation begins to make significant impacts on inner ear studies due to its ability to regulate genomic structure and activity in response to intracellular and environmental cues^14^. Epigenetic regulations including DNA methylation are important for cellular memory, which ensure heritable cell characteristics and functionality without changing DNA sequence^15,16^. DNA methylation is usually associated with silencing of gene expression^17–19^, of which the patterns are established by a sequence of methylation and demethylation processes during normal development^20^ and are maintained in differentiated cells throughout life^21^. It is suggested that DNA methylation is a reversible process that can be changed in response to exogenous signaling^21^. For instance, 5-azacytidine (5-aza), a derivative of cytidine, is able to inactivate DNA methyltransferases (Dnmts), which subsequently facilitates DNA demethylation and induces expression of silenced genes^22,23^. In a previous study, 5-aza was able to increase the expression of hair cell genes and proteins in mouse utricle sensory epithelia-derived progenitor cells *in vitro*^24^. However, it is unclear whether the same effects of 5-aza can be applied to *in vivo* and mature hair cell epithelium.

To explore ways of regenerating hair cells *in vivo*, deafen models are often generated in the laboratory to examine the regeneration effects of treatments. The methods of deafening mice include noise exposure, gene mutation and ototoxic drugs. These methods are usually selected according to the nature of the studies. Noise-induced hearing loss (NIHL) leads to various extent of deafness according to the noise intensity, frequency and exposure duration, which is often used in studies of environmental trauma induced hair cell degeneration^25^. Mutant animals with certain gene deletion are often utilized to explore the function of target genes such as *Atoh1* and Notch signaling genes^26,27^. Application of ototoxic drugs such as kanamycin showed relatively consistent consequences, with a typical pattern of OHC loss at the base of the cochlea, progressively moving apically, followed by IHCs degeneration^27^. Aminoglycosides are important as a second agent in the treatment of serious infections such as septicemia, nosocomial respiratory tract infections, complicated urinary tract infections and complicated intra-abdominal infections^28^. They are used for definitive combination of treatment of severe infections due to organisms such as Brucella spp and Listeria monocytogenes. Aminoglycosides are also a part of a multi-drug regimen for certain mycobacterial infections including drug resistant tuberculosis^29^. Moreover, aminoglycosides are critical first agents for the treatment of infant meningitis^30^. Due to their relatively wide applications, aminoglycoside-related ototoxicity is inevitable in clinics. Taken together, aminoglycoside-induced hearing loss model is suitable for hair cell regeneration research, which was selected in this study.

In this research, a combination of kanamycin and furosemide was used to damage adult mouse hair cells. Because OHCs are more sensitive to aminoglycoside^27^, we focused on the regeneration of OHCs in the present study. A DNA demethylation strategy was applied to deafened mature mouse cochleae to determine its role in OHC regeneration. The aim of this research is to determine whether the Dnmt inhibitor is able to stimulate the regeneration of cochlear OHC in the deafened mature mouse inner ear.

## Results

### Inner and outer hair cells were damaged in an ototoxicity mouse model

**Figure 1:**
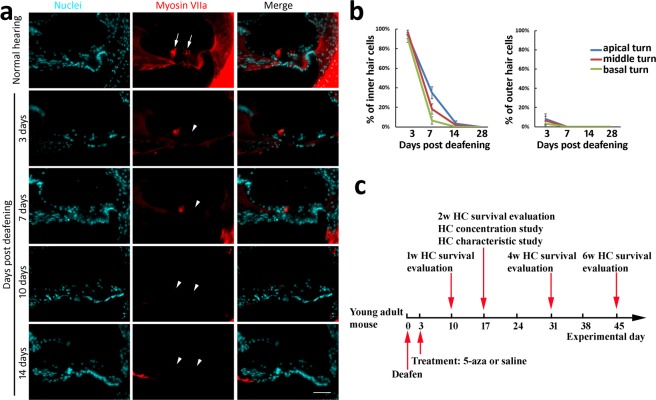
Loss of hair cell (HC) in chemically-damaged mature mouse inner ears. (**a**) Cochlear sections show HCs in normal hearing mice (arrows), whereas HCs are lost following chemical damage (arrowheads). (**b**) Quantitative study shows HC survival following chemical damage. In normal mouse cochlea, there are one row of IHCs and three rows of OHCs. Thus, the percentage of IHC survival is calculated by (number of IHCs/1) × 100%. The percentage of OHC survival is calculated by (number of OHCs/3) × 100%]. Scale bar: 50 µm. (**c**) The diagram shows the experimental design. The day of mice being deafened was set as experimental day 0. 5-aza or saline was injected into the mouse cochlea 3 days post deafening. Mice were followed up for 1–6 weeks. During one-week, two-week, four-week and six-week post-surgery, one group of animals were sacrificed, and their cochleae were cryosectioned to evaluate HCs. New HCs characterization and 5-aza concentration study were performed on animals two-week after 5-aza treatment.

Since aminoglycosides are important as a first and second agent in the treatment of many serious infections^28,30^, an aminoglycoside-induced ototoxicity model was generated by the application of kanamycin and furosemide in this study. Before 5-aza treatment, screening of IHC and OHC damage was performed to confirm degeneration of hair cells and determine the time for 5-aza treatment. The numbers of IHCs and OHCs were screened throughout the cochleae from 3 days to 14 days after deafening. Mouse cochlear samples of 3, 7, 10 and 14 days post deafening were collected throughout the cochleae and received immunofluorescence using hair cell specific anti-Myosin VIIa antibodies (Fig. 1a). As being consistent with other studies^31,32^, OHCs seemed to be more sensitive to ototoxicity, and were completely lost approximately 3–7 days post deafening, whereas IHCs were entirely damaged around 10–14 days after deafening (Fig. 1b). Because cell counting data demonstrated that over 90% OHCs had been damaged 3 days post deafening and also because we focused on regeneration of OHCs in this study, 5-aza was injected into mice 3 days post deafening. After determining the time for 5-aza injection, the timeline of the experimental procedures was designed and shown in Fig. 1c.

### Auditory hair cells were regenerated by Dnmt1 inhibition

**Figure 2:**
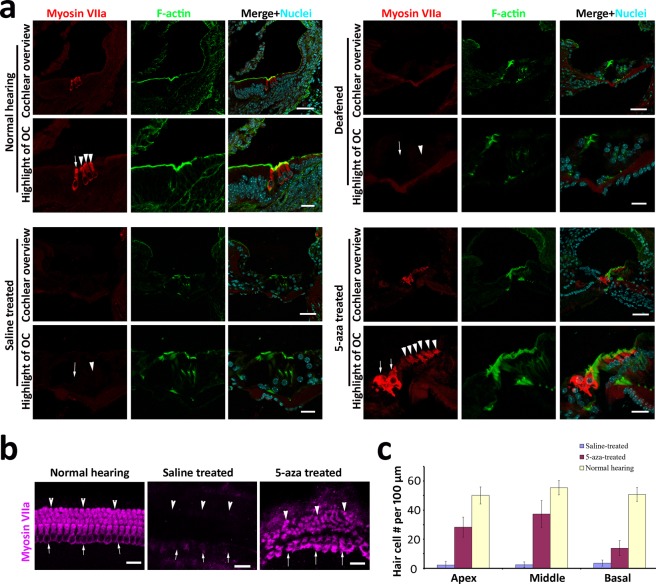
5-aza injection stimulates the generation of new HCs in a deafened mouse model. (**a**) Cochlear and organ of Corti (OC) images show expression of the hair cell protein Myosin VIIa (Myo7a) in the normal hearing mouse (arrow for IHC and arrowheads for OHCs), but not in saline-treated deafened mouse cochlea (arrow and arrowhead indicate IHC and OHC area respectively, without expression of HC protein Myosin VIIa). Two weeks post 5-aza injection into deafened mouse cochleae, cells express Myosin VIIa in the OC (arrows for IHCs and arrowheads for OHCs), suggesting new HC generation. Scale bar: 50 µm in cochlear section overview; 20 µm in OC highlight. (**b**) Basilar membrane surface preparations were obtained from mice two weeks after 5-aza or saline injection. Apical turn basilar membrane surface preparation shows Myosin VIIa-expressing HCs in normal hearing inner ear, but not in saline-treated deafened mouse inner ear. However, new HCs are found in 5-aza-treated deafened mouse inner ear. Arrows indicate the IHC area, whereas arrowheads suggest the OHC area. Scale bar: 20 µm. (**c**) Quantification of HCs in different cochlear turns in basilar membrane surface preparation two weeks after 5-aza or saline injection into deafened mice. One-way ANOVA was performed to compare the difference among the three groups, which showed a significant difference in all three cochlear turns (P < 0.01).

To understand whether the Dnmt1 inhibitor-5-aza stimulates the regeneration of OHCs, 5-aza or saline (control solution used to dissolve 5-aza) was injected into the left inner ear of chemically-deafened wildtype mature mice via the round window (Fig. 1c). Animals were maintained for two weeks after treatment to observe potential new hair cell regeneration (Fig. 1c). Double labeling of Myosin VIIa and F-actin was used to evaluate the presence of hair cells (HCs). The right cochleae (non-treated side) of the 5-aza treatment group were also kept and immunostained with Myosin VIIa to ensure the death of OHCs (data not shown). The cochlear cryosection of normal mice showed one IHC and three OHCs, double labeled by Myosin VIIa and F-actin (arrow and arrowheads in Fig. 2a). In the saline-treated group, the hair cell protein Myosin VIIa or F-actin immunostaining was not observed in the organ of Corti two weeks after saline injection (arrow and arrowheads in Fig. 2a). The deafened mouse cochlear section was also absent of Myosin VIIa or F-actin immunostaining (arrow and arrowheads in Fig. 2a). However, in the 5-aza group cells in the organ of Corti expressed both Myosin VIIa and F-actin (arrow and arrowheads in Fig. 2a). Basilar membrane surface preparation showed the number of Myosin VIIa-expressing HCs in the 5-aza group was greater than the saline-treated group, in which HCs were totally absent (arrow and arrowheads in Fig. 2b).

In the quantitative assay using whole mount study of counting HCs per 100 µm length of the basilar membrane, apex, middle and basal turns were separately quantified. In the apex turn of basilar membrane, 50 ± 6, 2 ± 3 and 28 ± 7 HCs per 100 µm were found in the normal hearing, saline- and 5-aza-treated groups respectively (Fig. 2c). In the middle turn, 55 ± 5, 2 ± 2 and 37 ± 9 HCs per 100 µm were found in the normal hearing, saline- and 5-aza-treated groups respectively (Fig. 2c). In the basal turn, 50 ± 5, 4 ± 2 and 14 ± 5 HCs per 100 µm were found in the normal hearing, saline- and 5-aza-treated groups respectively (Fig. 2c). One-way ANOVA was performed to compare the difference among the three groups. All three turns showed significant difference among the normal hearing, saline- and 5-aza-treated groups (Supplemental data Table 1). Within the 5-aza-treated group, there are significant differences among the apex, middle and basal turns (Supplemental data Table 1). Thus, it is concluded from the quantification of basilar membrane preparation that compared with the saline-treated group, there is a significant number of regenerated HCs in the 5-aza-treated group. In three cochlear turns, the middle turn showed best results of hair cell regeneration compared with apex and basal turns.

**Figure 3:**
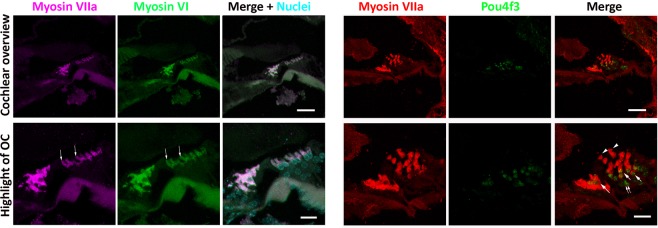
Newly-generated HCs express multiple hair cell proteins. New hair cell generation is observed two weeks following injection of 5-aza into the deafened mouse inner ear. These cells simultaneously express multiple hair cell specific proteins Myosin VIIa, Myosin VI and Pou4f3. Myosin VIIa and Myosin VI are found to co-express in the new HC-like cells (arrows). Myosin VIIa and Pou4f3 co-labeling is found in some cells (arrows), whereas some cells only express either Myosin VIIa (arrowheads) or Pou4f3 (double arrow). Scale bar: 50 μm in cochlear section overview; 20 μm in organ of Corti (OC) highlight.

Further immunofluorescence study showed that regenerated OHCs expressed other hair cell proteins including Myosin VI and Pou4f3 (Fig. 3). These cells simultaneously expressed several hair cell specific proteins in multiple labeling immunofluorescence (Fig. 3), indicating that these cells are likely HC-like cells. These data suggest that new HCs may be observed in the deafened mouse following 5-aza treatment.

### Determine the 5-aza concentration that is optimal for promoting new HC generation

**Figure 4:**
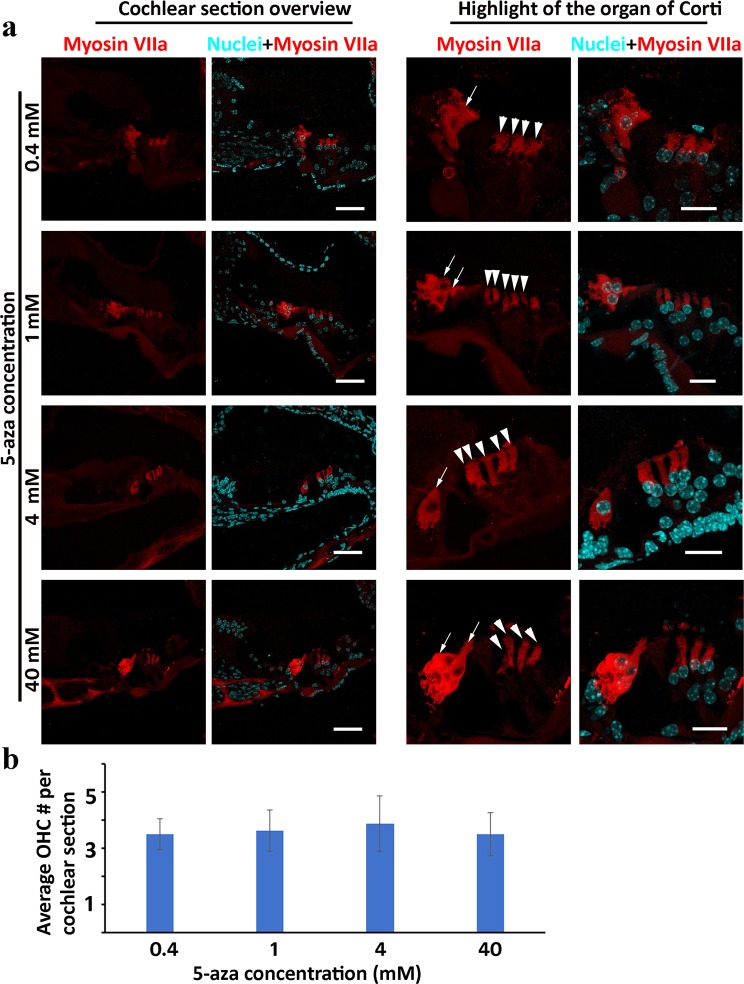
Generation of new hair cells by 5-aza is concentration dependent. (**a**) Deafened mice received 0.4, 1, 4 and 40 mM 5-aza injection and maintained for 2 weeks after injection. New Myosin VIIa-expressing HCs are observed in deafened mouse inner ear in 5-aza treated inner ear (arrows for the IHC area and arrowheads for the OHC area). Flask-like shape cells are observed in the IHC area (arrows), whereas columnar-shaped cells are found in the OHC area (arrowheads). Scale bar: 50 µm in cochlear section overview; 20 µm in organ of Corti (OC) highlight. (**b**) Quantification of the number of new OHCs in cochlear section in each 5-aza concentration group.

It was shown in our previous *in vitro* study that 40 µM was suitable for *in vitro* application of 5-aza while higher concentration caused cell death^24^. To determine the 5-aza concentration optimal for new HC generation *in vivo*, 0.4, 1, 4 and 40 mM 5-aza were injected into deafened mouse inner ears. New HCs were found in 12.5%, 37.5%, 75% and 25% of animals in these four groups two weeks post injection respectively (Fig. 4a,b). The chi-square statistic of the four-group comparison was 7.4667 and P = 0.058421, which was statistically insignificant. In a two-group comparison, the chi-square statistics of 0.4 vs 4 mM and 4 vs 40 mM groups were 6.3492 (P = 0.011743) and 4.0 (P = 0.0455) respectively, whereas the other two-group comparisons were statistically insignificant (P > 0.05; Supplemental data Table 2). In a quantification study to evaluate the average number of new HCs in each concentration, one-way ANOVA and Tukey *post hoc* test showed that there was no significant difference among the four concentration groups (P = 0.7415). Although not statistically significant, 4 mM group show a higher percentage of animals bearing new HCs and more HCs in cochlear sections (Fig. 4b, n = 8 mice in each group). Thus, 4 mM may be the concentration optimal for HC generation and was selected for the following studies.

### New HCs were survived for at least 6 weeks

**Figure 5:**
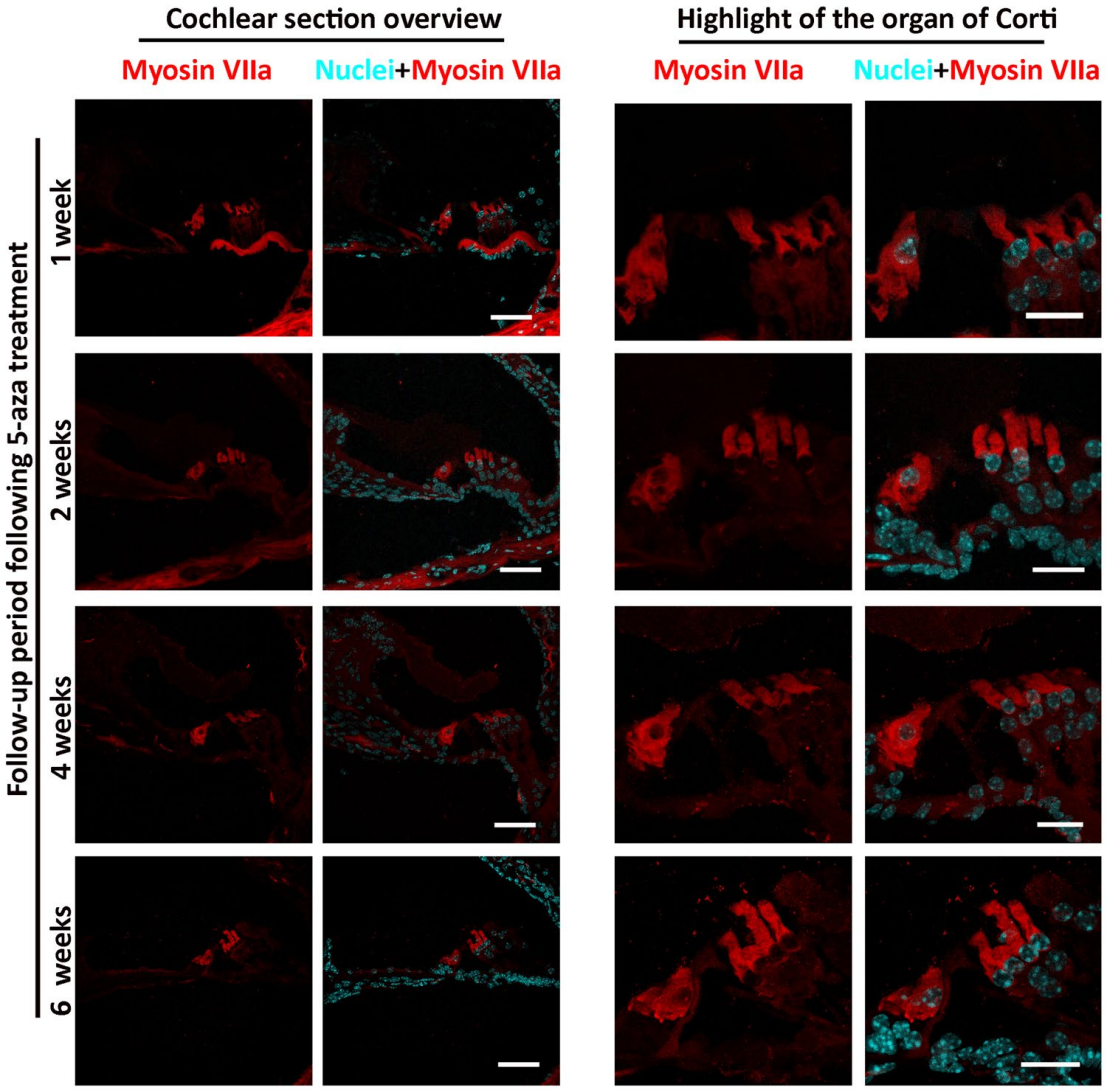
Survival of new HCs following 5-aza injection into deafened mouse inner ear. Following 4 mM 5-aza injection into the deafened mouse inner ear, new hair cell survival is seen for at least 6 weeks post 5-aza injection. Scale bar: 50 µm in cochlear section overview; 20 µm in organ of Corti highlight.

After determination of the optimal concentration of 5-aza, the survival time of new HCs was investigated by an additional set of animal experiment. Animals were deafened using the previous methods, and 4 mM 5-aza was applied to the deafened mice, which were kept for 1, 2, 4 and 6 weeks. New HCs were found in 37.5%, 75%, 50% and 62.5% of animals 1, 2, 4 and 6 weeks post injection respectively (Fig. 5, n = 8 mice in each group), suggesting that newly-generated HCs were able to survive for at least six weeks following 5-aza treatment.

### Normal hearing mice were not affected by 5-aza

**Figure 6:**
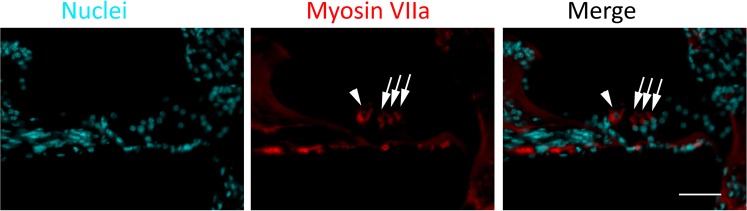
Normal hearing mice treated by 5-aza injection. After injection of 4 mM 5-aza into the cochleae of normal mice, the mice were followed up for two weeks. No changes of HCs are observed in cochlear sections. There are one row of IHC (arrowheads) and three rows of OHCs (arrows) in each cochlear section. Scale bar: 50 µm.

To explore the effect of 5-aza in normal hearing mice, 4 mM 5-aza was injected into the inner ear of young adult normal hearing mice (without deafening treatment). Two weeks after 5-aza injection, mice were euthanized. To be consistent with the majority of data in Figs 1–5, cochleae were continuously sectioned and labeled with Myosin VIIa to observe if there was any difference in the number of HCs between deafened and normal mice. All cochlear sections were collected for evaluation. There were one IHC and three OHCs in the section of normal mice with 5-aza treatment (arrows and arrowheads in Fig. 6), without significant changes in the number of HCs. These data indicate that 5-aza may not affect the number of HCs in normal hearing mice.

### Initial investigation of the mechanisms of new HCs generated from 5-aza treatment

**Figure 7:**
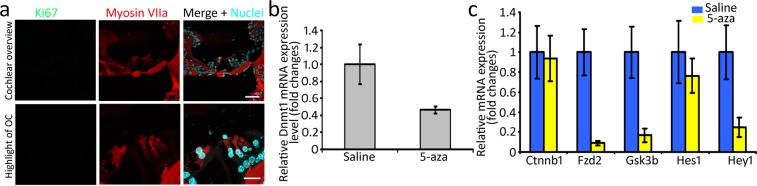
Initial mechanistic study of new HCs regeneration. (**a**) No Ki67 staining is observed in cochlear sections of mice two weeks after receiving 5-aza injection (Ki67 positive control in Supplemental Data Fig. S1). Scale bar: 50 µm in cochlear section overview; 20 µm in organ of Corti (OC) highlight. (**b**) Relative *Dnmt1* mRNA expression levels were measured in the saline- and 5-aza-injected deafened mice two weeks after injection. The results show decreased *Dnmt1* mRNA expression in the 5-aza group compared to the saline group. (**c**) Relative mRNA expression of Notch and Wnt signaling genes in saline- and 5-aza-treated deafened mice two weeks after injection. It is observed that expression of Notch signaling genes *Hes1* and *Hey1* as well as Wnt signaling genes *Fzd2* and *Gsk3b* are significantly downregulated, whereas *Ctnnb1* was not affected. Scale bar: 50 µm.

In experiments studying whether new HCs are generated via cell cycle reentry, Ki67 was used to label the sections of 5-aza-treated group two weeks after 5-aza injection. Ki67 immunostaining signal was not observed in the cochlear sections of 4 mM 5-aza treated mice (Fig. 7a, positive controls shown in Supplemental Data Fig. S1). Although negative Ki67 immunostaining cannot completely exclude the possibility of cell proliferation, it at least suggests that cell reentry was not identified at the observation time points or after hair cell differentiation. The origin of new HCs remains unclear, which may deserve an independent study.

Since 5-aza has been shown as a DNA methylation inhibitor by irreversibly binding to methyltrasferase^22,23^, it is logical to evaluate the expression of genes encoding DNA methyltransferase to understand whether the DNA methylation level is affected by 5-aza treatment. *Dnmt1*, which encodes DNA methyltransferase 1, was investigated in this study. Quantitative PCR was used to measure the level of *Dnmt1* mRNA in the control and 5-aza treated groups. The results show that compared to the control group, 5-aza treated mice exhibited significantly decreased *Dnmt1* expression (Fig. 7b). These data indicate possible DNA demethylation in the 5-aza group after 5-aza injection.

To evaluate whether Notch and Wnt signalings are involved in new HC generation, quantitative PCR was performed to determine the expression of Notch and Wnt signaling genes. It was observed that the expression of Notch signaling genes *Hes1* and *Hey1* as well as Wnt signaling genes *Gsk3b* and *Fzd2* was significantly reduced in the 5-aza group compared to the saline group^12,33^ (Fig. 7c). Down-regulation of Notch-related genes is consistent with the previous report^10^. However, reduced expression of Wnt signaling genes does not seem to be in lines with the data from the neonatal mice^11–13^, which deserves independent studies in the future.

### Evaluate the hair bundle of new HCs and initiate a protein-based functional assay

**Figure 8:**
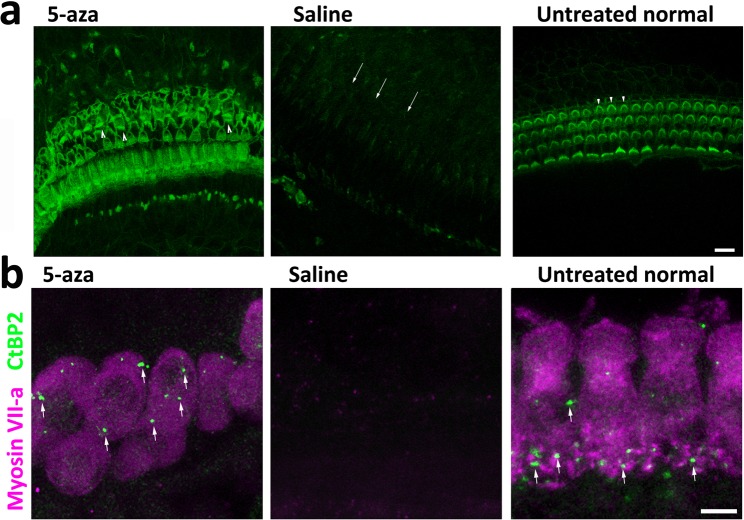
Hair bundle and presynaptic protein study of new HCs. (**a**) Basilar membrane preparation of mouse cochleae 2 weeks following 5-aza and saline treatment. Untreated normal hearing mice serve as a control (arrowheads showing W-shape of OHC hair bundles). Fluorescent phalloidin was used to label F-actin of HCs in the surface preparation. OHCs were absent in the saline group (arrows). Compared with the untreated group, the 5-aza group showed disorganized hair bundles (arrowheads). Scale bar: 10 µm. (**b**) Basilar membrane preparation of mouse cochleae 2 weeks following 5-aza and saline treatment. CtBP2 was used to label presynaptic vesicles of HCs in surface preparation. CtBP2 puncta are observed in untreated normal control and 5-aza groups (arrows), whereas they were not found in the saline group. Scale bar: 10 µm.

Comparing to the hair cell bundles of normal animals (Fig. 2), the bundles of newly-generated HCs seem abnormal and planar cell polarity seems to be lost. These observations raised our interests to investigate the hair bundle of new HCs. To further evaluate if hair cell bundles of new HCs are normal, F-actin labeling was performed in the surface preparations of normal, saline- and 5-aza-treated mice. The morphology of newly-regenerated HCs is shown in Fig. 8a. The hair bundle F-actin labeling in the OHC area of saline-treated mice was totally absent (arrows in Fig. 8a), indicating lack of hair bundle/OHCs in the saline group. In the 5-aza treated animals, abnormal hair cell bundles were observed (arrowheads in Fig. 8a) compared to the HCs in the normal animals. In addition, the planar cell polarity was lost in 5-aza treated mice with disorganized hair cell bundles. These abnormal hair cell bundles may indicate hair cell dysfunction. Notably, the F-actin labeling was missing in some of the OHC area, which also suggested impaired function of new HCs. Taken together, the abnormality of hair cell bundle F-actin labeling may indicate compromised functionality of new HCs.

To initiate the functional evaluation of new HCs, CtBP2 immunostaining was used to examine presynaptic vesicles. CtBP2 immunostaining showed that CtBP2 puncta were observed in the HCs of 5-aza treated mice and normal mice (arrows in Fig. 8b), whereas CtBP2 puncta expression was not detected in the saline-treated group (Fig. 8b). The positive CtBP2 staining suggests the possibility of presynaptic function of regenerated HCs.

## Discussion

In this study, the Dnmt1 inhibitor 5-aza was injected into chemically-deafened mature mouse cochleae. It was observed that the application of 5-aza stimulated the generation of new HCs in the chemically-deafened mouse cochlea. Immunofluorescence study revealed that new HCs expressed multiple hair cell specific proteins. New HCs were distributed in apical, middle and basal turns and were able to survive for at least six weeks. The optimal concentration of 5-aza to stimulate regeneration of HCs was 4 mM. The *Dnmt1* level was reduced in 5-aza treated mice by quantitative PCR. These results suggest that the Dnmt1 inhibitor facilitates regeneration of HCs in aminoglycoside-induced deafened mice.

5-aza was injected into the cochlea three days after deafening, at which only 3–8% surviving OHCs were observed in cell counting (Fig. 1a,b). The absence of Myosin VIIa immunoactivity at OHC region indicated that OHCs were completely damaged 3–7 days post deafening. In contrast, a number of HCs were found in the OHC region 1, 2, 4 and 6 weeks after 5-aza treatment (Figs 2–5). These data suggest that HCs located at the OHC area were likely newly-generated HCs. IHCs are not our interest of research in this study, over 90% of IHCs were observed at 3 days post deafening (Fig. 1a,b). IHC counting showed that a small proportion of IHCs survived for 3–7 days post deafening, but they were totally lost 14 days post deafening (Fig. 1b). Thus, surviving or recovery of original IHCs cannot be entirely excluded at 2-week follow-up. However, new HCs were found in the IHC area 4–6 weeks following 5-aza injection (Figs 2, 4 and 5), but not in the inner ears receiving saline. Additionally, the whole mount study by HC counting on basilar membrane demonstrated new HC generation in the 5-aza group (Fig. 2b). Therefore, these data suggest that 5-aza may promote regeneration of IHCs.

In the normal cochlea, HCs are arranged in one row of IHCs and three rows of OHCs (Fig. 2a). The regenerated OHCs in the 5-aza group showed irregular arrangement compared with the OHCs in the normal cochlea (Fig. 2a), suggesting disorganized growth of the new HCs. The cross-section immunofluorescence images demonstrated more than 3 rows of OHCs in some sections of 5-aza-treated cochleae (Fig. 2a), whereas the basilar membrane preparation in the 5-aza group showed a smaller number of OHCs than normal cochleae (Fig. 2c). This difference may be caused by the disorganization of new HCs, leading to uneven distribution of regenerated OHCs. Some areas showed more than three rows of OHCs, whereas some areas showed less than three rows of OHCs. Notably, the total number of OHCs in the 5-aza group was smaller than the normal animals. Therefore, these data suggest that the number of OHCs is not totally recovered by 5-aza treatment. In terms of IHCs, some of the images showed two cells instead of one, indicating that these extra IHCs may be newly-generated.

The hair cell specific markers used in this study include Myosin VIIa, Myosin VI, Pou4f3 and hair bundle-labeling F-actin. Here, it is shown that all these proteins were expressed in new HCs regenerated by the 5-aza application (Figs 2, 3). Merge images suggest that all these markers except for Pou4f3 were co-labeled in the newly-regenerated HCs. Possible reasons for the un-colocalized Myosin VIIa and Pou4f3 may include different cell developmental stages and/or different cryosection levels.

In the optimal concentration experiment, 0.4, 1, 4 and 40 mM 5-aza solutions were injected into the inner ear, and new HCs were found in 12.5%, 37.5%, 75% and 25% of animals in these four groups two weeks post injection respectively. These data indicate that the effect of 5-aza to regenerate new HCs is concentration relevant. At the concentration ranging from 0.4 to 4 mM, higher concentration causes more animals bearing with newly-regenerated HCs. However, the animal group receiving 40 mM 5-aza shows deceased regeneration of HCs. These results are consistent with the previous study that higher concentration of 5-aza decreased the viability of inner ear-derived stem cells^24^. Further studies are needed to determine the underlying mechanism of high concentration 5-aza toxicity to all cell types including HCs of the inner ear.

The surgery to administrate 5-aza into the mouse inner ear is through the round window injection. Quantification of HCs through the basilar membrane suggests the successful diffusion of 5-aza through the whole cochlea by the treatment (Fig. 2). Since 5-aza is injected via the round window at the basal cochlear turn, the basal turn should be the direct recipient of 5-aza solution and is expected to see robust HC regeneration. However, our observation reveals that the majority of OHCs are in the apex and middle turns (Fig. 2c). This result is consistent with the other studies of hair cell regeneration in mature and neonatal mice, with a remarkable number of HCs in the apical and middle turns^3,10^. It is, however, unclear why OHCs regeneration is less robust in the basal turn.

In the experiment testing the survival time of new HCs, HCs were observed from one week to six weeks post 5-aza treatment, indicating that these new HCs can survive for at least six weeks (Fig. 5). There are only 37.5% of animals found with regenerated HCs at one-week post 5-aza treatment, whereas at two weeks post-surgery 75% of animals are found with new HCs. Thus, the relatively smaller number of animals with new HCs in the one-week group suggests that one week may be the period that HCs regeneration is in the progress, so regeneration of HCs has not fully completed at one week after treatment. It was noticed that the percentage of animals showing new HCs declined from two-week post-surgery to four-week post-surgery, indicating that a proportion of regenerated HCs may be unable to survive for a longer time.

5-aza has been reported to function as a DNA methyltransferase inhibitor by irreversibly binding to methyltransferase^22,23^. To test whether 5-aza acted as a DNA methyltransferase inhibitor in triggering regeneration of HCs, expression of *Dnmt1* (encoding DNA methyltransferase 1) was examined. The reduced expression of *Dnmt1* in 5-aza treated mice comparing to control mice confirms the possible 5-aza-induced DNA demethylation (Fig. 7b). Additionally, downregulation of Notch and Wnt signaling genes suggests the involvement of Notch and Wnt signaling in the generation of new HCs (Fig. 7c), which should be investigated in the future. For the sources of new HCs, Ki67 immunostaining was performed to determine the presence or absence of cell proliferation. The absence of Ki67 immunostaining (Fig. 7a) suggests that cell reentry was not identified at the observation time points or after hair cell differentiation. The source and underlying mechanisms critical for new hair cell generation will be thoroughly explored in an independent study in the future.

It was noticed that the morphology of the hair cell bundles was abnormal in the 5-aza treated group, which may suggest compromised function of new HCs. Cryosection Myosin VIIa labeling (Figs 2, 4 and 5) and surface preparation F-actin labeling (Fig. 8a) confirm the abnormal hair bundle of new HCs. Comparing to the hair bundles in normal animals, the regenerated HCs exhibited disorganized bundles. Additionally, the planar cell polarity was missing in the regenerated HCs. These data suggest the compromised function of new HCs. CtBP2 is expressed in the synapses between the IHCs and spiral ganglion neurons. The expression of CtBP2 puncta in the IHC area of 5-aza treated mice indicates the formation of presynaptic vesicles in new HCs (Fig. 8b). It is noted that the CtBP2 puncta were not located at the basal lateral region as the native mature hair cell ribbon synapse. The misplacement may suggest that new HCs are in the developing stages. Interestingly, the number of puncta in the 5-aza group looks smaller than that in the normal mice (Fig. 8b), which also suggests possible compromised function of regenerated HCs. Due to these abnormalities of new HCs, a complete functional evaluation of new HCs using ABR was not performed in this study, which may be considered after these deficits are addressed.

In our previous report, 5-aza shows the capability to increase expression of hair cell genes and proteins in mouse utricle sensory epithelia-derived progenitor cells *in vitro*^24^. There is similarity of the previous and current studies including newly-generated HC-like cells expressing hair cell proteins and bundle-like structures. However, there are some differences between the previous report and current study. First, the current study investigates an *in vivo* model using deafened mice, whereas the previous paper explored an *in vitro* system using an inner ear stem cell line. Second, the previous study focused on the cell fate determination of stem cells following 5-aza application. The current study focuses on regeneration of HCs after 5-aza injection. Finally, in the previous paper, the sensory HC-like cells were derived from stem cells. In the current report, the sources of new HCs have yet to be fully-understood, which needs more efforts in the future.

In summary, this research investigates a novel epigenetic approach to regenerate HCs *in vivo* via DNA demethylation. The results of this study suggest that DNA demethylation may be sufficient to drive the regeneration of HCs in the mature mouse cochlea. The advantage of this epigenetic approach is that DNA sequences remain unchanged without integration of exogenous DNA sequence during treatment. Meanwhile, there are a number of unaddressed issues in this novel approach including the function of newly-regenerated HCs, the cell sources of new HCs and genetic changes of specific cell types in the cochlea, which deserve independent studies in the future.

## Material and Methods

### Animals

Young adult (four-six weeks old, either sex) wildtype Swiss Webster mice were included in the study. The care and use of animals have been approved by Wayne State University Institutional Animal Care and Use Committee. Methods were carried out in accordance with approved guidelines.

### Deafening

Kanamycin (1 g/kg body weight, s.c.) was injected subcutaneously followed by intraperitoneal injection of furosemide (300 mg/kg body weight, i.p.) after 25–30 minute. Pure tone baseline auditory brainstem responses (ABR) was applied to evaluate deafness of mice at frequencies of 8 kHz, 16 kHz, 24 and 32 kHz with stimulation of 10–90 dB SPL in 5–10 dB steps using the RP2.1 system (Tucker-Davis Technology; TDT). The TDT System 3 software was utilized for signal generation and response collection. ABR tests were performed on the same mice before deafening to record the baseline, and 3 days post-deafening to determine deafness. Mice with hearing threshold shift >40 dB SPL were qualified for the following studies. To quantify the HC survival after deafening, cochlear samples were harvested 3, 7, 10 and 14 days post deafening (n = 6 mice per group). Ten cochlear section samples (10 µm thickness) were collected at 50 µm intervals from each cochlea for immunostaining of hair cell specific anti-Myosin VIIa antibodies (refer to the Histology and immunofluorescence section). The number of IHCs and OHCs of each cochlear turn were counted, and the percentages of HC survival were determined against the number of HCs of normal hearing animals. In Fig. 1b, HC survival was determined by comparing IHCs and OHCs between the deafened mice and normal mice. The percentage of survived IHCs was calculated by (number of IHCs/1) × 100%. The percentage of survived OHCs was calculated by (number of OHCs/3) × 100%. For example, 1 IHC in the deafened mice will be shown as 100%, whereas 0 IHC in the deafened mice will be shown as 0%. For OHCs, 3 cells will be considered as 100%, 2 cells found will be counted as 67%, 1 OHC will be 33% of survival.

### Inner ear surgery

To determine the 5-aza concentration optimal for new HC generation, a series of 5-aza (0.4, 1, 4 and 40 mM) and saline (control, the solution used to dissolve 5-aza) were injected into 40 deafened mouse inner ears (n = 8 mice in each group). Under deep anesthesia, the left temporal bone was exposed and opened to visualize the basal cochlear turn and round window. A small catheter was inserted into the round window, by which 0.2 µl of 5-aza or saline solution was injected into the inner ear via a microsyringe (Hamilton). After the optimal concentration was determined, other groups of deafened mice were injected with the optimal 5-aza concentration and followed for 1, 2, 4 and 6 weeks to determine the survival of new HCs. At each follow-up point, 8 mice were included in both 5-aza and saline groups.

### Histology and immunofluorescence

At the end of the experiment, mice were euthanized, and cochlear samples were harvested and fixed by 4% paraformaldehyde. Cochleae were decalcified with 0.1 M ethylenediaminetetraacetic acid (EDTA, Sigma) for 5–7 days, followed by cryosectioning at 10 µm thickness. Some cochlear samples were used for basilar membrane surface preparation to quantify new HC regeneration in the whole mount study. For immunostaining, cochlear samples were treated with phosphate buffered saline (PBS) containing 5% normal donkey serum (Jackson ImmunoResearch) and 0.2% Triton X-100 (Sigma) for 30 min. Samples were incubated in primary antibodies at 4 °C overnight, followed by corresponding secondary antibody incubation at room temperature for 1–2 hr. The primary antibodies included: anti-Myosin VIIa (1:100; Developmental Studies Hybridoma Bank, DSHB, and Proteus Bioscience), anti-Myosin VI (1:100; Sigma), anti-Pou4f3 (1:200; Sigma), anti-CtBP2 (1:200; BD Bioscience) and anti-Ki67 (1: 200; Invitrogen). Secondary antibodies included Dylight-488, Cy3 and Dylight-647 conjugated antibodies (1:500; Jackson ImmunoResearch). 4,6-Diamidino-2-Phenylindole (DAPI; Invitrogen) was used to label all nuclei. Alexa Fluor 488- or 555-conjugated fluorescence phalloidin (Invitrogen) was used to label F-actin. Leica SPE confocal microscopy was used to capture all the images, except Fig. 1A and Fig. 6 (imaged by a Leica DM 2500 or Leica DMI 3000B epifluorescence microscope equipped with a Q-imaging cooled monochrome digital camera).

### Quantitative PCR (qPCR)

Total RNAs of the 5-aza group and saline group were extracted by TRI reagent (Sigma) method using manufacture’s methods. The total RNAs were converted into cDNA by the QuantiTect Reverse Transcription Kit (Qiagen) according to manufacturers’ protocols. A Bio-Rad CFX system was applied for qPCR using SsoAdvanced SYBR Green Supermix (Bio-Rad; n = 3). The mean of quantification cycle (Cq) was calculated by Bio-Rad CFX Manager software using a regression model. The relative expression levels of studied genes (primers listed in Supplement Table 3) were delta/delta Cq values normalized with internal control gene *Gapdh* using our published methods^34^.

### Statistics

In the 5-aza concentration study, the number of treated mice bearing Myosin VIIa-expressing HCs was counted in saline and 5-aza groups (n = 8 mice in each group). Data were shown in percentages of animals bearing Myosin VIIa-expressing cells. Chi-square statistical analysis was used to determine the concentration optimal for new HC generation. To determine the concentration optimal for HC regeneration, the number of HCs in cochlear sections was quantified. Cochlear sections of 5-aza treated group were collected. In each animal, eight cochlear sections at 50 µm interval were used to quantify the number of OHCs. Only the animals with regenerated HCs were included in quantification. One-way analysis of variance (ANOVA) was used to determine the significant difference. In the quantitative study of each cochlear turn, Myosin VIIa-expressing cells were counted in 100 µm length of basilar membrane of the normal hearing, saline- and 5-aza-treated deafened mice. Apex, middle and basal turns were separately quantified. The 100 µm of basilar membrane for counting HCs within each cochlear turn was randomly chosen. Labeling of Myosin VIIa with nuclei was used to count HCs. For quantification, animals without new HCs were not included. So only the animals with regenerated HCs were included in the HC quantification assay. The mean ± standard deviations were shown in the number of new HC quantification study. One-way ANOVA was used to determine the significant difference. P < 0.05 was considered as the criteria of statistical significance.

## Supplementary information

Supplementary Info

## Data Availability

All data generated or analyzed in this study are included in this published article (and its Supplementary Information Files).
