# Supplementary material for: Generation of new hair cells by DNA methyltransferase (Dnmt) inhibitor 5-azacytidine in a chemically-deafened mouse model
Source: Sci Rep. 2019 May 29;9:7997. doi: 10.1038/s41598-019-44313-0 (PMC6541592; doi:10.1038/s41598-019-44313-0)
Supplement: Supplementary file 1 — Supplementary Info [file 41598_2019_44313_MOESM1_ESM.pdf]

Generation of new hair cells by DNA methyltransferase (Dnmt) inhibitor 5-azacytidine in a chemically-deafened mouse model

Xin Deng, Zhenjie Liu, Xiaoyang Li, Yang Zhou and Zhengqing Hu*

Department of Otolaryngology-Head and Neck Surgery Wayne

State University School of Medicine

*Corresponding Author
Zhengqing Hu
Department of Otolaryngology – HNS, Wayne State University
550 E. Canfield St 258 Lande,
Detroit, MI 48201
United States
Phone: 313-577-0675

Email: zh@med.wayne.edu

Supplemental Figure

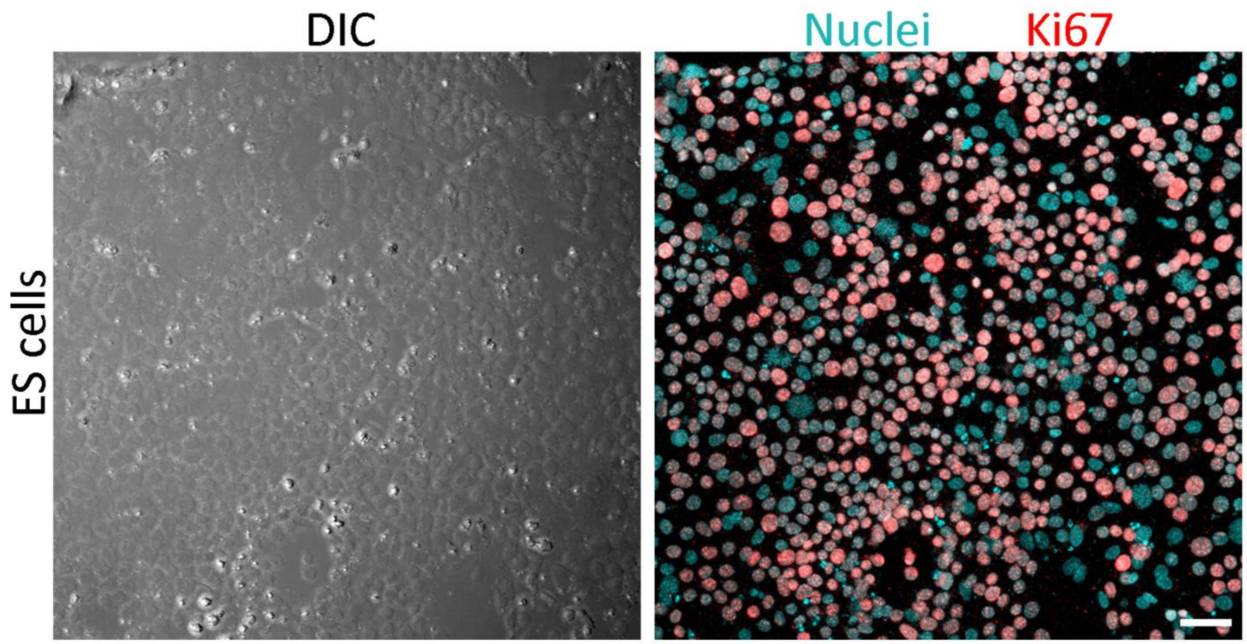

Figure S1. Positive control for Ki67 antibody.

Differential interference contrast (DIC) and immunofluorescence images show embryonic stem (ES) cells cultured for 3 days. After 3 days, the cells were fixed by 4% paraformaldehyde, follow with Ki67 immunostaining and DAPI nuclei staining. A proportion of ES cells were double-labeled by anti-Ki67 antibodies and the nuclei marker DAPI, suggesting cell proliferation. Scale bar: 25 µm.

Supplemental Table 1. One-way ANOVA statistical analysis of the number of hair cells in different cochlear turns

Groups	One-way ANOVA results		
Apex (normal hearing, 5-aza and saline)	$F_{(2,21)}=157.7$	$P<0.0001$	N=8
Middle (normal hearing, 5-aza and saline)	$F_{(2,21)}=153.4$	$P<0.0001$	N=8
Basal (normal hearing, 5-aza and saline)	$F_{(2,21)}=281.2$	$P<0.0001$	N=8
5-aza group (apex, middle and basal turn)	$F_{(2,21)}=3.5$	$P<0.0001$	N=8

Supplemental Table 2. Chi-square statistical analysis of the 5-aza concentration study

Groups (5-aza)	Chi-square statistic results
0.4 mM vs 4 mM	The chi-square statistic is 6.3492. The *p*-value is .011743.
1 mM vs 4 mM	The chi-square statistic is 2.2857. The *p*-value is .13057.
4 mM vs 40 mM	The chi-square statistic is 4. The *p*-value is .0455.
0.4 mM vs 1 mM	The chi-square statistic is 1.3333. The *p*-value is .248213.
0.4 mM vs 40 mM	The chi-square statistic is 0.4103. The *p*-value is .521839.
1 mM vs 40 mM	The chi-square statistic is 0.2909. The *p*-value is .589639.
4 groups	chi square statistic is 7.4667, p=0.058421

n= 8 mice in each group.

Supplemental Table 3. Primers for PCR

Gene	Forward	Reverse
Gapdh	GGCCGCATCTTCTTGTGCAGT	TTCTCGGCCTTGACTGTGCCGTT
Hes1	GATAGCTCCCGGCATTCCAAG	GCGCGGTATTTCCCCAACA
Hey1	CCGACGAGACCGAATCAATAAC	TCAGGTGATCCACAGTCATCTG
Fzd2	ATCCGCACCATCATGAAGCA	TACCGTGTAGAGCACCGAGA
Ctnnb1	GCAAATCATGCGCCTTTGCGGGAA	TGTGAACGTCCCGAGCAAGGATGT
Gsk-3b	CGAACTCCACCAGAGGCAAT	AAGAGTGCAGGTGTGTCTCG
Dnmt1	GTCGGACAGTGACACCCTTT	TGGGTTTCCGTTTAGTGGGG
